# Supplementary material for: Diversity and Distribution of Macrofungi in Protected Mountain Forest Habitats in Serbia and Its Relation to Abiotic Factors
Source: J Fungi (Basel). 2022 Oct 13;8(10):1074. doi: 10.3390/jof8101074 (PMC9605310; doi:10.3390/jof8101074)
Supplement: Supplementary file 1 [file jof-08-01074-s001.zip › jof-1869784-supplementary.pdf]

Tabela S1. Macrofungal species recorded in protected mountain forest habitats in Serbia

SPECIES	FG	Number of records					I year				II year			III year				F
		P1	P2	P3	P4	P5	VI	VII	VIII	IX	VI	VII	VIII	VI	VII	VIII	IX	
**total number of species***		53	48	59	110	80	32	38	53	30	33	19	26	69	71	31	54	
**ASCOMYCOTA**																		
Bisporella citrina (Batsch) Korf & S.E. Carp.	Lig				1										P4			1
Caloscypha fulgens (Pers.) Boud.	Ter					1								P5				1
Disciotis venosa (Pers.) Arnould	Lig	1									P1							1
Helvella calycina Skrede, T.A. Carlsen & T. Schumach.	Lig				1									P4				1
Hypoxylon fragiforme (Pers.) J. Kickx f.	Lig				1			P4										1
Kretzschmaria deusta (Hoffm.) P.M.D. Martin	Lig				1								P4					1
Lachnellula resinaria (Cooke & W. Phillips) Rehm	Lig			1											P3			1
Neobulgaria pura (Pers.) Petr.	Lig				2	1									P4,5		P4	3
Peziza phyllogena Cooke	Ter	1					P1											1
Spathularia flavida Pers.	Lig		2						P2						P2			2
Vibrissea truncorum (Alb. & Schwein.) Fr.	Lig				3		P4				P4			P4				3
Xylaria carpophila (Pers.) Fr.	Lig	2			1									P1,4	P1			3
Xylaria hypoxylon (L.) Grev.	Lig	1	1		3	1		P4,5					P4		P1,2,4			6
**BASIDIOMYCOTA**																		
Agaricus sylvicola (Vittad.) Peck	Ter		1											P2				1
Agrocybe praecox (Pers.) Fayod	Lig	1													P1			1
Amanita battarrae (Boud.) Bon	Mik			5	1			P3	P3			P3			P3,4	P3		6
Amanita excelsa (Fr.) Bertill.	Mik				1				P4									1
Amanita fulva Fr.	Mik		1												P2			1
Amanita rubescens Pers.	Mik		1			2	P2		P5						P5			3
Bjerkandera adusta (Willd.) P. Karst.*	Lig	1		1	6	1	P3,4	P4	P4		P1		P4	P4	P4,5			9
Boletus edulis Bull.	Mik				2										P4		P4	2
Boletus fechtneri Velen.	Mik					1							P5					1
Boletus luridiformis Rostk.	Mik				2	1							P4,5				P4	3
Boletus subtomentosus L.	Mik				1												P4	1
Caloboletus calopus (Pers.) Vizzini	Mik				3			P4					P4		P4			3
Calocera cornea (Batsch) Fr.	Lig				4	1		P4						P4	P4,5		P4	5
c (Fr.) Fr.	Lig		1					P2										1
Calocera viscosa (Pers.) Fr.*	Lig		1	3	4	1		P4	P5				P3	P4	P4	P3	P2,3,4	9
Cantharellus cibarius Fr.*	Mik		1	1	1	2		P4,5	P2,P5	P3								5
Cerioporus varius (Pers.) Zmitr. & Kovalenko*	Lig	8	2	7		1	P1,3	P1,3	P1,2,3	**P3**	P3	P1,3	P1,2,5	P1	P1,3	P3	P1	19
Chalciporus piperatus (Bull.) Bataille	Mik					1								P5				1

Climacocystis borealis (Fr.) Kotl. & Pouzar	Lig					1											P5	1
Clitocybe gibba (Pers.) P. Kumm.	Ter			2		1			P5							P3	P3	3
Clitocybe nebularis (Batsch) P. Kumm.	Ter			1													P3	1
Coprinellus micaceus (Bull.) Vilgalys, Hopple & Jacq. Johnson	Lig			1	2						P3			P4			P4	3
Coprinellus xanthothrix (Romagn.) Vilgalys, Hopple & Jacq. Johnson	Ter	3							P1				P1	P1				3
Coprinopsis lagopus (Fr.) Redhead, Vilgalys & Moncalvo	Ter	1												P1				1
Cortinarius croceus (Schaeff.) Gray	Mik			1													P3	1
Cortinarius evernius (Fr.) Fr.	Mik				2			P4		P4								2
Cortinarius flexipes (Pers.) Fr.	Mik				1					P4								1
Cortinarius sanguineus (Wulfen) Gray	Mik				1					P4								1
Cortinarius semisanguineus (Fr.) Gillet	Mik					1			P5									1
Craterellus tubaeformis (Fr.) Quél.	Mik				2					P4							P4	2
Crepidotus variabilis (Pers.) P. Kumm.	Lig			1					P3									1
Dacrymyces capitatus Schwein.	Lig				1							P4						1
Dacrymyces chrysospermus Berk. & M.A. Curtis	Lig					1			P5									1
Dacrymyces stillatus Nees	Lig		1	1				P2									P3	2
Daedalea quercina (L.) Pers.	Lig					1										P5		1
Daedaleopsis confragosa (Bolton) J. Schröt.	Lig				1		P4											1
Datronia mollis (Sommerf.) Donk	Lig				1								P4					1
Echinoderma asperum (Pers.) Bon	Ter	1														P1		1
Entocybe nitida (Quél.) T.J. Baroni, Largent & V. Hofst.	Ter				1												P4	1
Entoloma serrulatum (Fr.) Hesler	Lig					1									P5			1
Exidia thuretiana (Lév.) Fr.	Lig				1									P4				1
Flammulaster carpophilus (Fr.) Earle ex Vellinga	Ter	2					P1							P1				2
Flammulaster muricatus (Fr.) Watling	Lig	1					P1											1
Flammulina velutipes (Curtis) Singer	Lig			1													P3	1
Fomes fomentarius (L.) Fr.	Lig			2	1	2			P3,5		P3		P5	P4				5
Fomitopsis pinicola (Sw.) P. Karst.	Lig			2	3	7	P4,5	P5	P5	P5	P3	P5	P3,4,5	P4,5				12
Galerina hypnorum (Schrank) Kühner	Lig				1									P4				1
Galerina marginata (Batsch) Kühner	Lig				1						P4							1
Ganoderma applanatum (Pers.) Pat.	Lig				8	5	P4,5	P4	P4,5	P4,5	P5	P4	P4,5	P4			P4	13
Geastrum quadrifidum DC. ex Pers.	Ter		1						P2									1
Gloeophyllum odoratum (Wulfen) Imazeki	Lig				3									P4	P4	P4		3
Guepinia helvelloides (DC.) Fr.	Lig				1										P4			1
Gymnopus androsaceus (L.) Della Magg. & Trassin.*	Ter		2	3	1	2	P2,3,5	P2,3,5							P3,4			8

Gymnopus confluens (Pers.) Antonín, Halling & Noordel.	Ter	1								P1								1
Gymnopus dryophilus (Bull.) Murrill*	Ter	2	1	3		2	P3	P5	P3					P1,2	P1,3,5			8
Gymnopus foetidus (Sowerby) P.M. Kirk	Ter	3			1		P1				P1			P1	P4			4
Gymnopus perforans (Hoffm.) Antonín & Noordel.	Ter			1						P3								1
Gyroporus castaneus (Bull.) Quél.	Mik				1										P4			1
Gyroporus cyanescens (Bull.) Quél.	Mik			1									P3					1
Hemimycena lactea (Pers.) Singer	Ter				2	1			P5					P4	P4			3
Hericium coralloides (Scop.) Pers.	Lig					3				P5				P5	P5			3
Hydnum repandum L.	Mik		1			1			P2	P5								2
Hydropus subalpinus (Höhn.) Singer	Ter	1		2			P3							P1	P3			3
Hygrophorus erubescens (Fr.) Fr.	Mik			1					P3									1
Hymenopellis radicata (Relhan) R.H. Petersen*	Lig	8	2	2	1		P1	P1	P2	P1	P1		P2,3	P1	P1	P1,3,4	P1	13
Hypholoma capnoides (Fr.) P. Kumm.	Lig		1												P2			1
Hypholoma fasciculare (Huds.) P. Kumm.*	Lig	3	1	3	2	1			P1		P1,3	P3		P2,5	P1	P3,4	P4	10
Hypholoma lateritium (Schaeff.) P. Kumm.	Lig				1	1							P5	P4				2
Laccaria amethystina Cooke	Mik					1			P5									1
Laccaria laccata (Scop.) Cooke	Mik		3	1						P3	P2			P2	P2			4
Laccaria longipes G.M. Muell.	Mik				1					P4								1
Lactarius vellereus (Fr.) Fr.	Mik		3					P2								P2	P2	3
Lactarius volemus (Fr.) Fr.	Mik		2						P2							P2		2
Lentinellus micheneri (Berk. & M.A. Curtis) Pegler	Lig				1											P4		1
Lycoperdon echinatum Pers.	Ter	1				1				P5					P1			2
Lycoperdon perlatum Pers.*	Ter		1	2		1									P2	P3	P3,5	4
Chlorophyllum rhacodes (Vittad.) Vellinga	Ter			1												P3		1
Marasmius bulliardii Quél	Ter	6			1	1	P1,5	P4				P1		P1	P1	P1	P1	8
Marasmius rotula (Scop.) Fr.	Ter	2	2		2	2	P1,2	P1,2,5							P4,5		P4	8
Megacollybia platyphylla (Pers.) Kotl. & Pouzar	Lig	4	1			2	P1		P1		P1,2	P4		P1		P4		7
Micromphale perforans (Hoffm.) Antonín & Noordel.	Ter				1	1		P4,5										2
Mucidula mucida (Schrad.) Pat.	Lig			1	1	2				P5	P3,5						P4	4
Mycena acicula (Schaeff.) P. Kumm.	Lig	1			1									P4	P1			2
Mycena arcangeliana Bres.	Lig				1						P4							1
Mycena epipterygia (Scop.) Gray	Lig				2			P4			P4							2
Mycena galericulata (Scop.) Gray*	Lig	1	1	2	1	1	P3							P1,2,5	P4		P3	6
Mycena galopus (Pers.) P. Kumm.	Ter		1		3	2		P4,5						P2,4	P4,5			6
Mycena haematopus (Pers.) P. Kumm.	Lig	1			1									P1,4				2

Mycena inclinata (Fr.) Quél.	Lig				1				P4									1
Mycena laevigata Gillet	Lig					1					P5							1
Mycena leptocephala (Pers.) Gillet	Lig				1										P4			1
Mycena maculata P. Karst.	Lig				1					P4								1
Mycena mirata (Peck) Sacc.	Ter			1	1	1			P3						P4,5			3
Mycena pelianthina (Fr.) Quél.	Ter	3		1							P1			P1	P1,3			4
Mycena polygramma (Bull.) Gray	Lig				1	1					P4				P5			2
Mycena pura (Pers.) P. Kumm.*	Ter	3	2	3	1	1	P1,3		P3		P2			P1,4	P1,2,3,5		P4	11
Mycena renati Quél.	Ter				1						P4							1
Mycena rubromarginata (Fr.) P. Kumm.	Ter		1		1	1	P2	P4						P5				3
Mycena sanguinolenta (Alb. & Schwein.) P. Kumm.*	Ter		1	1	3	1		P4							P2,3,4,5		P4	6
Mycena stylobates (Pers.) P. Kumm.	Ter	1	1			2		P5	P5					P1,2				4
Mycena viridimarginata P. Karst.	Lig				1						P4							1
Mycena vitilis (Fr.) Quél.	Ter			1	4	1		P4				P4		P4,5	P3		P4	6
Mycetinis alliaceus (Jacq.) Earle ex A.W. Wilson & Desjardin	Ter			3	7	4	P3	P4	P4,5		P4,5	P4		P4,5	P3,4,5		P3,4	14
Mycetinis scorodonius (Fr.) A.W. Wilson & Desjardin	Ter		1			2	P5	P2,5										3
Neoboletus erythropus (Pers.) C. Hahn	Mik					1			P5									1
Paxillus involutus (Batsch) Fr.	Mik				1												P4	1
Phaeocollybia lugubris (Fr.) R. Heim	Ter			1													P3	1
Phallus impudicus L.	Ter	5	3				P2	P2	P1			P1			P1	P1,2	P1	8
Phloeomana speirea (Fr.) Redhead	Lig				1							P4						1
Pholiota flammans (Batsch) P. Kumm.	Lig			1													P3	1
Picipes badius (Pers.) Zmitr. & Kovalenko	Mik				1					P4								1
Pleurocybella porrigens (Pers.) Singer	Lig				1	2				P5							P4,5	3
Plicaturopsis crispa (Pers.) D.A. Reid	Lig	1		1					P3					P1				2
Pluteus cervinus (Schaeff.) P. Kumm.*	Lig	1		1	2	3					P3,5	P1	P5	P4	P5		P4	7
Pluteus griseoluridus P.D. Orton	Lig				1												P4	1
Pluteus phaeocyanopus Minnis & Sundb.	Lig					1											P5	1
Pluteus romellii (Britzelm.) Sacc.	Lig			2							P3				P3			2
Polyporus arcularius Rostk.	Lig	1												P1				1
Cerioporus squamosus (Huds.) Quél.	Lig	2							P1								P1	2
Porphyrellus porphyrosporus (Fr. & Hök) E.-J. Gilbert	Mik			2		1									P5	P3	P3	3
Psathyrella candolleana (Fr.) Maire	Lig	2				1		P1	P5						P1			3
Pseudohydnum gelatinosum (Scop.) P. Karst.	Lig			1	2	4		P5	P3,4,5					P5	P5		P4	7
Radulomyces molaris (Chaillet ex Fr.) M.P. Christ.	Lig					1								P5				1
Rickenella fibula (Bull.) Raithelh.	Ter				3	1						P4		P4	P4,5			4
Rickenella swartzii (Fr.) Kuyper	Ter				2							P4					P4	2

Russula amethystina Quél.	Mik					1									P5			1
Russula cyanoxantha (Schaeff.) Fr.	Mik	1			2	1		P4	P5			P4			P1			4
Russula firmula Jul. Schäff.	Mik					1							P5					1
Russula foetens Pers.	Mik		2					P2							P2			2
Russula fuscorubroides Bon	Mik				1					P4								1
Russula grisea Fr.	Mik		1								P2							1
Russula mustelina Fr.	Mik			1									P3					1
Russula nigricans Fr.	Mik				1					P4								1
Sarcodon scabrosus (Fr.) P. Karst.	Mik			1					P3									1
Schizophyllum commune Fr.	Lig				1									P4				1
Sparassis crispa (Wulfen) Fr.	Lig				1	1				P4,5								2
Stereum hirsutum (Willd.) Pers.	Lig	3	1		1				P1				P1	P1	P2,4			5
Strobilomyces strobilaceus (Scop.) Berk.	Mik			1													P3	1
Suillus grevillei (Klotzsch) Singer	Mik					1											P5	1
Trametes gibbosa (Pers.) Fr.	Lig				3		P4			P4			P4					3
Trametes hirsuta (Wulfen) Lloyd	Lig	1															P1	1
Trametes pubescens (Schumach.) Pilát	Lig	1					P1											1
Trametes trogii Berk.	Lig			1							P3							1
Trametes versicolor (L.) Lloyd	Lig	5	2						P1			P1		P1	P1,2	P1	P2	7
Tremella mesenterica Retz.	Lig				1												P4	1
Trichaptum abietinum (Pers.) Ryvarden	Lig					2	P5						P5					2
Tricholomopsis rutilans (Schaeff.) Singer	Lig		2											P2		P2		2
Tubaria furfuracea (Pers.) Gillet	Lig	1												P1				1

Legend: FG – functional group; F – frequency of occurence; * including data for the specimens identified to the genus level

Note: Specimens identified to the genus level (5 among Ascomycota and 79 among Basidiomycota) are excluded from the table (represented list of species), for easier visibility. They can be obtained on request.
